# The COVID-19 Pandemic and Its Effects on Mental Health—A before, during, and after Comparison Using the U.S. Census Bureau’s Household Pulse Survey

**DOI:** 10.3390/ijerph21101306

**Published:** 2024-09-30

**Authors:** Aarnav D. Shah, Christina Laternser, Priyamvada Tatachar, Priscilla Duong

**Affiliations:** 1 Lake Forest Academy, Lake Forest, IL 60045, USA; 2 Ann & Robert H Lurie Children’s Hospital of Chicago, Chicago, IL 60611, USA; claternser@luriechildrens.org (C.L.); ptatachar@luriechildrens.org (P.T.); pduong@luriechildrens.org (P.D.); 3 The Division of Neurology, Department of Pediatrics, Feinberg School of Medicine, Northwestern University, Chicago, IL 60208, USA; 4 Department of Psychiatry & Behavioral Sciences, Feinberg School of Medicine, Northwestern University, Chicago, IL 60208, USA

**Keywords:** COVID-19, anxiety, depression, mental health, U.S. population

## Abstract

Background: Although significantly increased mental health concerns were noted globally during the first year of the COVID-19 pandemic, much less is known about the mental health trends during the COVID-19 recovery period. We aimed to compare current anxiety or depression rates to those before and during the first year of the pandemic and to evaluate demographic differences. Methods: We analyzed Household Pulse Survey data prospectively collected from a representative U.S. population sample. We compared the anxiety or depression rates from the first pandemic year (04/2020–05/2021) and recovery period (06/2023–03/2024) from the national cohort and demographic subgroups using two-sided paired *t*-tests and regression analyses and compared these to pre-pandemic (01/2019–12/2019) rates using one-sided *t*-tests. Results: The national estimates for anxiety or depression improved during the recent COVID-19 recovery period as compared to the first year (29.5 ± 5.5 vs. 37.6 ± 3.1; *p* < 0.0001) but did not return to the pre-pandemic benchmark (29.5% vs. 10.8%; *p* < 0.001). Higher rates were noted in younger individuals aged 18–29 years (*p* < 0.0001), in individuals with less than a high school diploma (*p* < 0.0001), or with disabilities (*p* < 0.0001). Non-Hispanic Asians reported the lowest rates (*p* < 0.0001), and no significant gender differences were noted. Conclusion: The U.S. population’s mental health concerns have improved since the first year of the pandemic but remain above pre-pandemic benchmarks. Certain demographic subgroups are at higher risk, indicating the need for targeted health care and economic policy interventions to address these disparities.

## 1. Introduction

The coronavirus disease 2019 (COVID-19) pandemic caused a global rise in mental health issues [1,2]. The COVID-19 pandemic had a widespread negative impact on society outside of lives lost. The public health measures to prevent the spread of COVID-19 employed during the pandemic restricted social interaction. This social isolation thereby raised loneliness rates, led to job and income insecurity for many households, and increased individuals’ concerns about health and mortality [3]. Chronic and acute stress exposure are known risk factors for anxiety and depression in population studies [4,5]. In the United States of America, the same time period at the beginning of the pandemic also had additional stressors on the society, such as economic decline that started in the fall of 2019, the presidential election in 2020, and social unrest and protests against interpersonal racism. To evaluate the national effect of this pandemic-related stress, the U.S. Census Bureau’s Household Pulse Survey (HPS) collected data on self-reported symptoms of anxiety or depression starting from April 2020 [6], from which we can glean the effects of the pandemic on a subset of mental health concerns during the peak and recovery period of the pandemic. Prior to the pandemic, a 2019 NHIS [7] report used a similar survey, which showed that 10.8% of the U.S. population reported symptoms of anxiety or depression [7], serving as a pre-pandemic benchmark value for self-reported anxiety or depression rates to be used in comparison with the HPS data. Multiple studies have reported high levels of anxiety and depression at the beginning of the pandemic and increasing rates from the beginning of the pandemic for the first year of the pandemic, with rates being almost 6-fold higher compared to pre-pandemic levels [8,9,10,11,12]. The U.S. government has officially declared that the COVID-19 pandemic is no longer considered a public health emergency as of spring of 2023^8^, lifting any special provisions that allow people to return to their pre-pandemic state of existence. However, there are limited data on the current status of mental health, and it is not clear if anxiety or depression rates have lessened in this “recovery period” since spring 2023. Understanding and comparing the current state of mental health in a nationally representative sample and different demographic subgroups is necessary to guide health care and economic policies and funding allocation to effectively target the national mental health crisis left in the wake of the COVID-19 pandemic.

Our primary aim was to analyze current anxiety or depression rates during the “recovery period” (06/2023–03/2024) and compare them to the rates from the first year of the pandemic (04/2020–05/2021) and the pre-pandemic era (2019), and to see if the current rates have converged to the pre-pandemic rates. Our secondary aim was to evaluate the current rates of anxiety or depression in the following demographic subgroups: age, sex, gender, educational attainment, race and ethnicity, and disability status.

## 2. Materials and Methods

We analyzed cross-sectional data on the rates of anxiety and depression in a representative sample of the U.S. population published by the Centers for Disease Control.

Data source: The U.S. Census Bureau, in cooperation with several other federal agencies, developed a nationally representative online survey known as the Household Pulse Survey (HPS) to understand the development of anxiety and depression rates starting from April 2020, and it is currently ongoing. The HPS utilized the Census Bureau’s Master Address File as a source of a randomly selected sample. Respondents were administered a Modified (using two questions from the standardized questionnaire) Patient Health Questionnaire-2 (PHQ-2) to assess the respondent’s depression level. These two questions included “How often have you been bothered by having little interest or pleasure in doing things?” and “How often have you been bothered by feeling down, depressed, or hopeless?”. A modified (using two questions from the standardized questionnaire) Generalized Anxiety Disorder-2 (GAD-2) questionnaire was administered that included these two questions: “How often have you been bothered by the following problems: Feeling nervous, anxious, or on edge?” and “how often have you been bothered by the following problems: Not being able to stop or control worrying?”. The items index how often symptoms were experienced in the past two weeks (0, not at all; 3, nearly every day). Responses were summed to create a depression score and anxiety score, with totals of 3 or greater interpreted as clinically significant [13,14]. The data were collected every seven days, from 30 April 2020 to 21 July 2021, and every two weeks after August 2021 onwards. The data were accessed in June 2024. The current study analyzed the HSP data from Phases 1, 2, 3, and part of 3.1 administered between April 2020 and March 2021 as “first-year data” and compared it to the HSP data from phases 3.8, 3.9, 3.10, and 4.0 administered between April 2023 and March 2024, denoted as the “recovery period” data. The pooled sample size for the first-year data was 1,144,405 responses from 30 total surveys and the pooled sample size for the “recovery period” was 750,799 responses from 11 surveys administered during that period that represented about 7% of the response rates on average.

The HPS data were compared with the pre-pandemic benchmark data published by the NHIS 2019 survey, which used a similar survey methodology [7]. The 2019 NHIS report used the exact same questions as the Household Pulse Survey of PHQ-2 and GAD-2 to gauge the respondents’ mental health state.

Outcome variables: We reported the rates of anxiety or depression as a combined measure of mental health by utilizing the weekly or bimonthly anxiety or depression rates as the main outcome variables. The HPS data, a time series dataset, was aggregated to display the percentage of adults having anxiety or depression. The first-year HPS data from April 2020 to May 2021 were grouped as “first-year data” and compared with the “recovery period” data from April 2023 to March 2024.

Statistical analysis: The average rates of anxiety or depression reported during the recovery period were compared with first-year average rates using two-tailed *t*-tests. Using one-tailed *t*-tests, we also compared the first year and recovery period rates with the pre-pandemic benchmark rates from the NHIS 2019 report. The rates during the recovery period were further analyzed by comparing the demographic subgroups of age, sex, gender, educational attainment, race and ethnicity, and disability status. The results from these comparisons are shown in Table 1. To better understand how demographic subgroups compared to each other and between the first year of COVID-19 and the recovery period, we implemented an ordinary least squares regression model with robust standard errors. The ordinary least squares technique was used as it is considered to obtain the best linear unbiased estimator and because there was no control population available for statistical comparison.

## 3. Results

The average rates of anxiety and depression during the recovery period in the national cohort and demographic subgroups have improved as compared to the first year of the pandemic but remain elevated as compared to the pre-pandemic benchmarks (Table 1).

### 3.1. National Estimates of Anxiety or Depression

**Figure 1:**
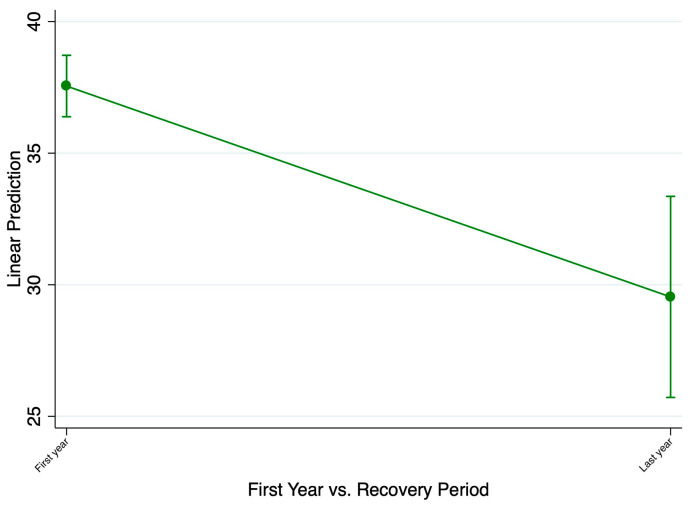
Comparison of the average rates of anxiety or depression between the first year of the pandemic and the current year representing the post-COVID-19 period.

The national estimate for the current rates of anxiety or depression during the recovery period after spring 2023 in the representative U.S. population is reported to be 29.5%, which is improved as compared to the beginning of the pandemic rates of 37.6% (Figure 1). It is important to note that linear probability models using ordinary least squares regression are accurate in comparing the average rates but not at extremes. Because our data do not deal with extreme values, it is statistically valid to use ordinary least squares regression for our analyses. Although the rates have improved in more recent years, the current rates are still elevated as compared to the pre-pandemic rates of 10.8% reported by the NHS 2019 data, as shown in Table 1. A similar trend is seen with rates improved since the first year of the pandemic but not completely recovered since the pre-pandemic data within demographic subgroups divided by sex, age, education level, race, disability status, sexual orientation, or gender identity.

### 3.2. Effects of Demographic Factors

#### 3.2.1. Age

**Figure 2:**
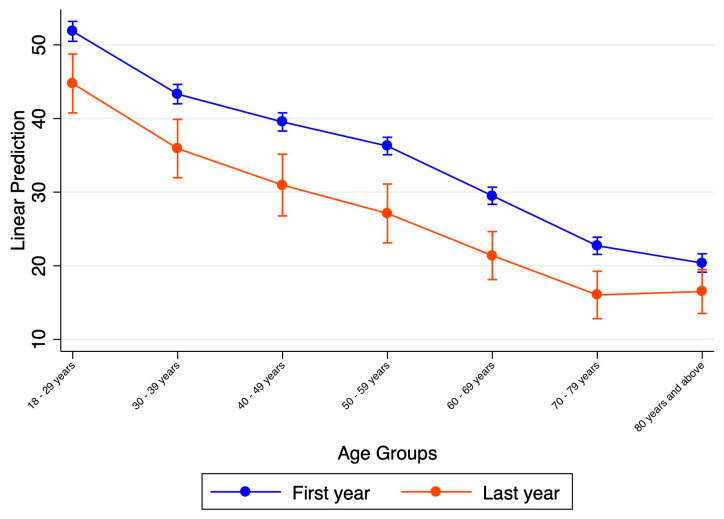
Comparison of the average rates of anxiety or depression in different age groups between the first year of the pandemic and the current year representing the post-COVID-19 period.

Anxiety or depression rates within different age groups were noted during the first year of the pandemic and the recovery period (Figure 2) and an improvement in mental health was shown within all the age-based subgroups. The rate of decline is consistent across groups as the slopes representing this decline are not statistically different. Even though the rates have improved for the individual subgroups, significant variations exist within the subgroups, with younger Americans reporting the highest symptoms of anxiety or depression during the first year of COVID-19, and this trend continued in the most recent year of the data. Further, these results suggest that the oldest Americans included in the survey (80 years and older) experienced slightly higher rates of anxiety or depression rates as compared to the 70–79-year-old group during the recovery period. Otherwise, there is no significant difference in the rate of decline for anxiety or depression rates across the age groups.

#### 3.2.2. Educational Attainment

**Figure 3:**
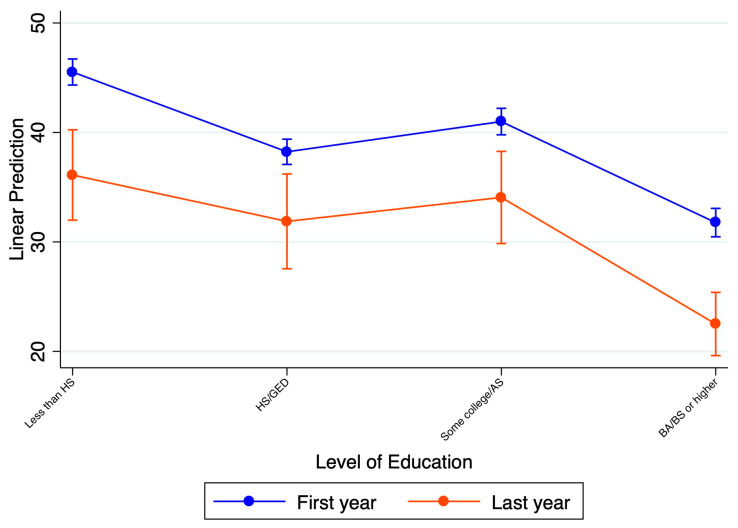
Comparison of the average rates of anxiety or depression between the first year of the pandemic and the current year representing the post-COVID-19 period between different educational attainment groups.

The education attainment subgroup showed a difference between the first year of COVID-19 and the recovery period across all subgroups, as displayed in Figure 3. However, the relationship between more years of education and anxiety or depression rates is non-linear. Specifically, those with a bachelor’s degree or higher had significantly lower rates than the other subgroups. However, the groups with some college degrees reported higher rates than those with high school GEDs and no college education. There is no significant statistical difference in the rate of decline across the educational attainment groups.

#### 3.2.3. Race and Ethnicity

**Figure 4:**
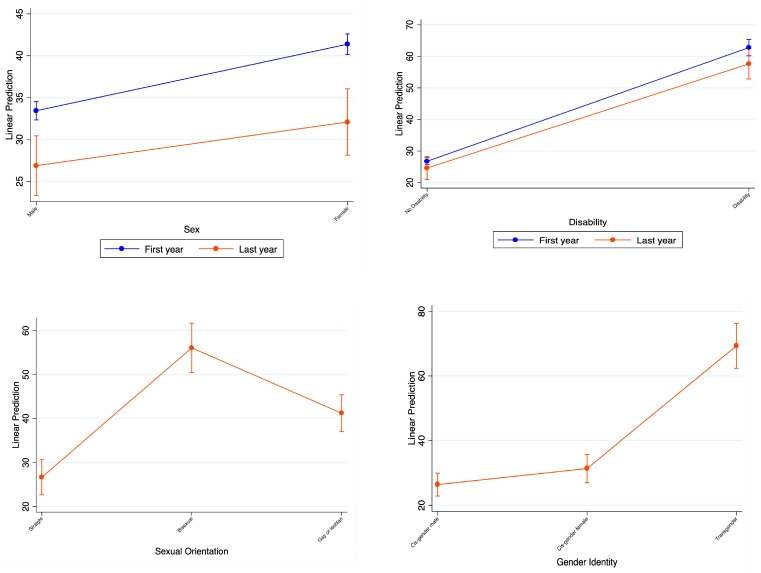
Comparison of the average rates of anxiety or depression between the first year of the pandemic and the current year representing the post-COVID-19 period within subgroups based on sex, disability status, sexual orientation, and gender identity.

Figure 4 shows a comparison of the average rates of anxiety or depression between the first year of the pandemic and the current year representing the post-COVID-19 period between different race and ethnicity subgroups.

In the subgroup analysis of race and ethnicity, people who identify as Other or multi-racial or ethnically as Hispanic had higher rates of anxiety and depression in the first year of COVID-19 as well as in the recovery period as compared to those who identify as white or black. However, anxiety or depression rates have reduced for all races and ethnicities in the recovery period as compared to the first year of COVID-19, and the rates of decline are similar for each subgroup; significant differences in the rates within the subgroups persist with continued higher rates reported by the Hispanic and “Other” communities. Although black Americans reported higher rates of symptoms during the first year of the pandemic as compared to white Americans, these two subgroups now have similar rates during the recovery period. There is no statistically significant difference in the rate of decline in anxiety or depression rates across race and ethnicity.

#### 3.2.4. Sex, Disability Status, Sexual Orientation, and Gender Identity

While analyzing sex differences, the first year of COVID-19 showed slightly higher anxiety or depression rates than the recovery period. However, the differences between male and female sex assigned at birth are minimal, albeit statistically significantly different across time periods and sex. In the analysis of the subgroups based on reported disability or no disability, their subgroup rates for the first year vs. the last year are minimal. However, the group with disabilities reported significantly higher rates during the first year (36% more) as well as the recovery period (33% more) as compared to those without reported disabilities. Additionally, we do not see a marked improvement in anxiety or depression in people with disabilities across the two periods. For sexual orientation and gender identity, only recovery period data were available. People who identify as bisexual or transgender had markedly higher anxiety or depression rates than the other two comparison groups. People who identify as heterosexual had statistically significantly lower anxiety or depression rates than people who identify as bisexual, gay, or lesbian. There was no statistically significant difference between those who identified as cis-gender male and those who identified as cis-gender female.

## 4. Discussion

Our study showed that during the current COVID-19 pandemic recovery period, the U.S. population reported improvements in the rates of anxiety or depression as compared to the rates reported during the first year of the pandemic. However, these rates have not completely recovered in comparison to the pre-pandemic period. Additionally, our study showed that significant differences exist in these measures of poor health in certain demographic subgroups. Specifically, those in the younger age group of 18–29 years old, those with lower educational attainment, Hispanic or “other” race, or individuals with disability report significantly higher rates of poor mental health symptoms as compared to their peers. At least, we observe that the rate of decline is relatively consistent across groups and time periods, and anxiety or depression rates are falling for most groups at around the same rate as the other comparison groups. Most importantly, marginalized groups, specifically those with disabilities, those who identify as bisexual, gay/lesbian, transgender, multi-racial, Hispanic ethnicity, or those with lower educational levels disproportionally face significantly higher anxiety or depression rates.

General prevalence of anxiety or depression: Multiple prior studies have reported that the rates of anxiety or depression increased significantly within the U.S. population during the initial COVID-19 pandemic [8,9,10,11,12]. This increase was likely influenced by multiple factors compounding the uncertainties of that time period, including the shifting political environment with the presidential election in 2020, weakening economy and increasing inflation rates, widespread national social unrest and protests against individual racism, and worsening global climate crises. In addition to these multiple risk factors, this period was punctuated by mandated isolation from family members and a lack of consistent and clear governmental messaging regarding health policies.

The rates were not only higher in 2020 as compared to 2019 but they also continued to increase for the initial year [8,9]. This increase in mental health concerns was also noted in a study evaluating data from nine countries reporting significantly increased primary care visits for mental health issues, primarily anxiety and depression, indicating a global mental health decline at the beginning of the pandemic. [15]. Our study shows encouraging data that these rates of anxiety and depression have improved in recent years. However, it is still concerning that the current rates remain higher compared to the pre-pandemic benchmark data, and therefore, we have only achieved partial recovery. A study reporting data from the 2017 U.S. National Health and Wellness Survey [16] indicated that depression is associated with lower health-related quality of life and work productivity, as well as higher healthcare utilization. Therefore, the continued elevated rates of poor mental health in the U.S. population noted by our study are anticipated to have a major economic burden and increased healthcare resource needs as a nation if the rates do not continue to fall and come to the baseline in the near future.

Age as a factor: Our study found that the younger age group continues to experience a much higher prevalence of anxiety or depression as compared to the other age groups, with the 18–29 years age group reporting three-fold higher rates (45%) as compared to the >70-year-olds (15%). Similar findings of a younger age being a risk factor for anxiety and depression and an increased need for mental health services have been reported by prior studies evaluating young Americans and college students [17,18]. Several factors can explain why young adults may have felt more anxious or depressed. For example, a survey of college students showed that a loss of household income, food insecurities, and housing insecurities were the strongest predictors of anxiety and depression in this age group [19], and these circumstances were common during the pandemic. Another study evaluating college students found that pandemic-associated loneliness caused boredom and repetitive negative thinking, leading to depression after COVID-19-related campus closures [20]. Along with these indicators of poor mental health in young adults, it is not surprising that a survey conducted in June 2020 found that the 18–24 years age group had the highest suicidal ideation, with one out of four having seriously considered suicide in the past 30 days [3]. Younger adults have shown a heightened responsivity to stressors [21]. It is possible that a perceived lack of control over the circumstances combined with an unstable social safety net and concern for personal and global future and productivity contributed heavily to the higher rates among young adults. These young adults are still in their developmental transition period, and uncertainty around educational and job opportunities due to the pandemic is likely to have a much greater effect on this younger age group as compared to the middle-aged and older adults, who may have more financial and social stability to lean on during the pandemic [18].

Self-reported measures report that even after the recovery from the COVID-19 pandemic, about 45% of young adults reported symptoms of anxiety or depression. It may be the case that depression or anxiety are going undiagnosed in older individuals because the modified GAD-2 and PHQ are measuring self-reported well-being. Self-reported measures report on how the individual perceives themself to be. Hence, we cannot rule out that anxiety and depression levels in older individuals are higher than the self-reported levels [22]. There is an important need to address this issue to prevent the future economic effects of this mental health crisis in young adults.

Education level as a factor: Our study showed that anxiety and/or depression rates improved at all education levels in the recovery period. However, there are still significant differences between those with a bachelor’s degree or higher and those with lower educational attainment. Our findings are similar to previously published research evaluating a large (125,000 Norwegian participants) population-based HUNT study looking at adults over the age of 24 years. This study revealed that lower education levels were significantly correlated with anxiety and depression [23]. A study from France also showed that those with lower education levels had a higher risk of anxiety or depression. Finally, a U.S. study using the HPS data from April 2020 to May 2021 corroborated our results; considerably higher rates of anxiety and depression were observed for those with less than a bachelor’s degree [9]. It is likely the case that those with at least a bachelor’s degree either have more savings in case of an emergency, have higher salaries that can more easily offset periods of potential employment uncertainty, or can more easily find a new job in case of being laid off, reducing overall stressors; however, this is only an assumption.

Race/Ethnicity as a factor: Hispanic and Latino and people identifying as mixed race were more likely to have higher anxiety or depression rates during the first year of COVID-19 as well as during the recovery period. People identifying as non-Hispanic blacks observed the greatest drop in anxiety or depression rates. However, no race or ethnicity fell to pre-pandemic rates. The impact of COVID-19-related discrimination towards historically marginalized racial–ethnic groups on psychological distress and sleep disturbances in the U.S. has been well documented [24]. In comparison, Asian Americans are less likely to utilize mental health services due to the stigma around mental health issues [25]. A study from 2003 attributes these differences to a disparity in health insurance and greater health burdens [26]. Although we could not test this in this study, there is a high correlation between educational attainment and race/ethnicity, and the effects of race/ethnicity and educational attainment may be interrelated [27]. In addition to the existing uncertainties of this time, a crisis point in racial and social injustice and the attention to pre-existing police violence was magnified by the incident of George Floyd on 25 May 2020, during the height of the COVID-19 pandemic. This incident led to nationwide protests and riots, influencing the already tenuous mental health crises in the country. Additionally, there were targeted attacks on Asians, specifically of Chinese origin, which created the “Stop Asian Hate” movement. These events undoubtedly caused a rise in anxiety within the U.S.

Disability status as a factor: Our study found that responders with disabilities reported more than double the rates of anxiety or depression as compared to those without in both the first year of COVID-19 and the recovery period. Since we lack finer data points parsing the source of anxiety or depression at an individual level, it is hard to isolate the exact reason for the higher levels among individuals with disabilities. Notably, the anxiety or depression rates were already high in this group in the first year of the pandemic. In this period, 57% of those with a disability reported symptoms of anxiety or depression. In comparison, a pre-pandemic study evaluating the mental health of those with disabilities reported a 33% prevalence of these symptoms [28], suggesting that the current rates, even after the pandemic recovery, have not returned to the pre-pandemic baseline. The same study found that 7.2% of those without disabilities reported mental health issues, indicating a 1:4.6 ratio between those without and those with disabilities.

In comparison, the current rates in our study indicate the ratio being 1:2.3, mainly due to a higher rate of increase in the non-disability group at 24.5% currently vs. 7.2% prior to the pandemic. It is important to note that disability is a broad term, spanning both physical and mental disabilities. We were not able to account for these differences. Those with mental disabilities are more likely to have higher rates of anxiety or depression than the general population. An analogous argument can be made for those with physical disabilities.

Differences between males and females: Females reported higher rates of anxiety or depression than males during the first year of COVID-19 and the recovery period. This is a similar finding reported by a French study noting that women had significantly higher rates of anxiety or depression than men at the beginning of the pandemic [29]. However, females did show a slightly greater decrease in anxiety or depression rates in the recovery period than males. In our study, the rates of anxiety or depression did not differ significantly in the recovery period, which is encouraging. However, the rates for males and females were still markedly higher in the first year of COVID-19 and the recovery period compared to the pre-pandemic era.

Sexual orientation and gender identity: The HPS did not have data available for the sexual orientation or gender identity demographic subgroups during the first year of COVID-19. However, we observed differences in the demographic subgroups for the data available during the recovery period. People identifying as heterosexual had much lower anxiety or depression rates than people identifying as bisexual or gay/lesbian. Further, those identifying as transgender had markedly higher rates of anxiety or depression during the recovery period than cis-gender males or cis-gender females.

Limitations of the study:

A multitude of factors and changes in both the micro and macro environments influence mental health. However, we could not control other potential demographic factors that may play a role, such as income level, loss of income, marital status, etc. Furthermore, within our race and ethnicity data, there is no specification for certain subgroups such as “Non-Hispanic Asian”, which had the lowest rates of mental illness, and “non-Hispanic multiple or other races”, which had the highest rates. These groups can be very diverse, and the specification of subgroups within these categories may reveal more findings. We also did not have data available for sexual orientation and gender identity during the first year of COVID-19, so a comparison across time is difficult to make. We also do not have more granular data available on the racial and ethnic or sex breakdown for disability to further analyze disability.

Additionally, the HSP had non-unique respondents, meaning that different randomly selected households took each survey. According to the American Community Survey, respondents to the HPS were more likely to be women, non-Hispanic white, and with higher education. Therefore, the HPS data may not completely represent the U.S. population [30]. Weights for each group were not provided, so we could not re-weight the sample to correct the potential imbalance.

## 5. Conclusions

Although anxiety or depression rates are falling, they have not nearly fallen to pre-pandemic levels; in some demographic subgroups, they have hardly fallen at all. Although the pandemic has been declassified as a health emergency, lingering effects remain relative to anxiety or depression rates. The stress of isolation, the possibility of unemployment, increased pressure for those who maintained their jobs throughout the pandemic amidst massive layoffs, long-term health effects from getting COVID-19, and the extreme loss of family and community members have left a lasting impact on anxiety or depression rates in the U.S. This does not factor in the additional strain faced by many households during the recovery period: rising interest rates, rising cost of living, rising health care expenses, rising food costs, rising mortgage and interest rates, etc. In addition to this, access to mental health care has been suboptimal, specifically for high-risk populations across the country. This study only scratches the surface of a deeper issue: the long-term effects of COVID-19 on mental health are astronomically larger for all Americans, especially those in marginalized demographics. This illustrates that much work needs to be done to assist the populace in overcoming the current challenges in the hope of improving the rates of anxiety and depression, at the very least, to the pre-pandemic rates. It is clear that the U.S. is still experiencing a mental health crisis even after COVID-19; policies targeting the sources of stress for Americans are needed to lower the continued anxiety or depression experienced by all.

## Figures and Tables

**Table 1:** Comparison of average percentages of anxiety or depression rates reported during different time periods in relationship to the COVID-19 pandemic.

	Rates of Anxiety or Depression during the First Year of COVID-19 (04/2020–05/2021)	Rates of Anxiety or Depression during the Recovery Period (04/2023–03/2024)	Comparison of First-Year vs. Recovery Period **	Comparison of Pre-Pandemic vs. First-Year Period *	Comparison of Pre-Pandemic vs. Recovery Period *
	Mean (S.D.)	Mean (S.D.)	*p*-Valueα	*p*-Valueα	*p*-Valueα
National estimate	37.6 (3.1)	29.5 (5.5)	<0.0001	<0.0001	<0.0001
Sex					
Male	33.4 (2.9)	26.9 (5.3)	<0.0001	<0.0001	0.0001
Female	41.4 (3.3)	32.1 (5.8)	<0.0001	<0.0001	<0.0001
Age					
18–29	51.8 (3.7)	44.8 (6.0)	<0.0001	<0.0001	<0.0001
30–39	43.3 (3.6)	35.9 (5.9)	<0.0001	<0.0001	<0.0001
40–49	39.5 (3.3)	31.0 (6.3)	<0.0001	<0.0001	<0.0001
50–59	36.3 (3.2)	27.1 (6.0)	<0.0001	<0.0001	0.0001
60–69	29.5 (3.2)	21.4 (4.9)	<0.0001	<0.0001	0.0005
70–79	22.7 (3.2)	16.0 (4.8)	<0.0001	<0.0001	0.0178
80+	20.4 (3.4)	16.5 (4.5)	0.0056	<0.0001	0.0085
Education level					
<HS diploma	45.5 (3.2)	36.1 (6.1)	<0.0001	<0.0001	<0.0001
HS diploma or GED	38.2 (3.1)	31.9 (6.4)	0.0002	<0.0001	<0.0001
Some college or associate’s degree	41.0 (3.3)	34.1 (6.3)	<0.0001	<0.0001	<0.0001
Bachelor’s degree or higher	31.8 (3.5)	22.5 (4.3)	<0.0001	<0.0001	0.0001
Race/Ethnicity					
Black ***	40.2 (3.1)	28.9 (6.5)	<0.0001	<0.0001	0.0001
White ***	35.8 (3.2)	28.8 (5.3)	<0.0001	<0.0001	<0.0001
Asian ***	31.8 (3.9)	22.0 (5.7)	<0.0001	<0.0001	0.0009
Other/multiple ***	46.4 (3.4)	39.3 (5.7)	<0.0001	<0.0001	<0.0001
Hispanic	42.6 (3.2)	32.6 (6.4)	<0.0001	<0.0001	<0.0001
Disability status					
No	26.8 (0.9)	24.6 (4.7)	0.5549	0.0259	0.0001
Yes	62.8 (2.2)	57.6 (6.1)	0.2951	0.0190	<0.0001
Sexuality ^†^					
Straight		26.7 (5.5)			0.0001
Bisexual		56.1 (7.6)			<0.0001
Gay or lesbian		41.1 (5.7)			<0.0001
Gender ^†^					
Cis-gender male		26.4 (4.8)			<0.0001
Cis-gender female		31.4 (5.9)			<0.0001
Transgender		69.3 (9.4)			<0.0001

HS = high school; GED = General Education Development. * One-sided *t*-test. We used 10.8% for all because we did not have a breakdown by demographic subgroups. ** Two-sided paired *t*-test comparing the first year (during the pandemic) and the last year of data (recovery period). *** Non-Hispanic. ^†^ Data not available for the first year of the pandemic. α Because of the large number of responders in each group, any small differences may have a statistically significant *p*-value.

## Data Availability

This study analyzed data collected by U.S. Census Bureau and published by the Center for Disease Control and Prevention accessed on 5 June 2024, Mental Health-Household Pulse Survey-COVID-19 (cdc.gov).
